# Androgen receptor contributes to repairing DNA damage induced by inflammation and oxidative stress in prostate cancer

**DOI:** 10.55730/1300-0152.2667

**Published:** 2023-10-11

**Authors:** Bilge DEBELEÇ BÜTÜNER, Nurşah ERTUNÇ HASBAL, Elif İŞEL, Dirk ROGGENBUCK, Kemal Sami KORKMAZ

**Affiliations:** 1 Department of Pharmaceutical Biotechnology, Faculty of Pharmacy, Ege University, İzmir, Turkiye; 2 Department of Bioengineering, Cancer Biology Laboratory, Faculty of Engineering, Ege University, İzmir, Turkiye; 3 Department of Chemistry, Simon Fraser University, Burnaby, BC, V5A 1S6, Canada; 4 Faculty of Health Sciences Brandenburg, Brandenburg University of Technology Cottbus-Senftenberg, Senftenberg, Germany; 5 Faculty Environment and Natural Sciences, Brandenburg University of Technology Cottbus-Senftenberg, Senftenberg, Germany

**Keywords:** Prostate cancer, γH2AX, inflammation-induced carcinogenesis, reactive oxygen species, androgen receptor, NKX3.1

## Abstract

**Background:**

Androgen deprivation therapy remains the first-line therapy option for prostate cancer, mostly resulting in the transition of the disease to a castration-resistant state. The lack of androgen signaling during therapy affects various cellular processes, which sometimes paradoxically contributes to cancer progression. As androgen receptor (AR) signaling is known to contribute to oxidative stress regulation, loss of AR may also affect DNA damage level and the response mechanism in oxidant and inflammatory conditions of the prostate tumor microenvironment. Therefore, this study aimed to investigate the role of AR and AR-regulated tumor suppressor *NKX3.1* upon oxidative stress-induced DNA damage response (DDR) in the inflammatory tumor microenvironment of the prostate.

**Materials and methods:**

Intracellular reactive oxygen species (ROS) level was induced by either inflammatory conditioned media obtained from lipopolysaccharide-induced macrophages or oxidants and measured by dichlorodihydrofluorescein diacetate. In addition to this, DNA damage was subsequently quantified by counting gH2AX foci using an immunofluorescence-based Aklides platform. Altered expression of proteins function in DDR detected by western blotting.

**Results:**

Cellular levels of ROS and ROS-induced DNA double-strand break damage were analyzed in the absence and presence of AR signaling upon treatment of prostate cancer cells by either oxidants or inflammatory microenvironment exposure. The results showed that AR suppresses intracellular ROS and contributes to DNA damage recognition under oxidant conditions. Besides, increased DNA damage due to loss of NKX3.1 under inflammatory conditions was alleviated by its overexpression. Moreover, the activation of the DDR mediators caused by AR and NKX3.1 activation in androgen-responsive and castration-resistant prostate cancer cells indicated that the androgen receptor function is essential both in controlling oxidative stress and in activating the ROS-induced DDR.

**Conclusion:**

Taken together, it is concluded that the regulatory function of androgen receptor signaling has a vital function in the balance between antioxidant response and DDR activation.

## 1. Introduction

### 1.1. Reactive oxygen species (ROS)-induced oxidative DNA damage in prostate cancer (PCa)

Oxidative stress results from either increased reactive oxygen/nitrogen species generation or an inadequate response of antioxidant mechanisms in cells. ROS-induced oxidative damage upon tumorigenesis might be related to different cellular alterations from expressional changes to structural alterations of proteins involved in DNA repair, apoptosis, and cell cycle (Khandrika et al., 2009). ROS cannot only trigger oxidative damage to various cellular components and impair cellular flows but also regulates redox signaling, thereby inducing highly specific acute or chronic alterations in the cellular environment (Ehsani et al., 2021). Therefore, an adequate response against ROS production is crucial to activate damage repair and maintain cell viability. Considering that a combination of factors mentioned above indicates the damage repair capacity of a cell in an organism, a cellular damage level that exceeds the repair capacity necessitates the evasion of the cell to provide an evolutionary benefit to others. This is achieved by maintaining the balance between the apoptotic and antiapoptotic pathways. Thus, ROS may induce DDR or apoptotic/necrotic cell death depending on the level of oxidative stress (Finkel 2003), and the threshold of this stress tolerance is maintained by the genetic background.

ROS plays an increasingly important role in the malignant transformation of normal prostate epithelial cells and the progression of PCa. Increased ROS contributes to cancer progression by acting as a DNA damaging agent, and its moderately elevated levels, which act as secondary messengers, cause somatic DNA mutations to interfere with various signaling pathways of oncogenic transcription factors, described in PCa studies (Khandrika et al., 2009).

### 1.2. Interaction of inflammation and ROS in DNA damage induction and repair activation

Oxidative stress is associated with several pathological conditions, including inflammation and infection. An inflammatory tumor microenvironment enhances the accumulation of ROS, leading to DNA damage and causing angiogenesis and metastasis in the progression of cancer (Finkel 2003; Reuter et al., 2010). Cytokines and growth factors also produce ROS as secondary messengers, particularly in the TNFα signaling pathway (Ushio-Fukai 2009; Morgan and Liu 2011). TNFα-induced ROS increases p53 activation by elevating the oxidative stress and DNA damage levels (Liu et al., 2008). DNA double-strand break (DSB) is a type of ROS-induced DNA damage characterized by histone H2A.X phosphorylation. It is a variant form of H2A, and it is required for ATM-dependent checkpoint-mediated cell cycle arrest and DSB-DNA repair activation (Yuan et al., 2010). In general, ionizing radiation and UV light result in the rapid phosphorylation of H2A.X at the Ser139 residue by PI3K-like kinases, including ATM, ATR, and DNA-PK (Rogakou et al., 1998; Burma et al., 2001) at the site of DNA damage (Yuan et al., 2010). However, ROS-mediated DSB has also been observed in some tumor cells, which needs further investigation.

### 1.3. Androgen signaling and the androgen-regulated tumor suppressor NKX3.1 in PCa

Androgens regulate the development, growth, and maintenance of prostate function. Therefore, a link between androgen levels and PCa has also been observed. The action of androgens in the cell is controlled by AR. Androgens are represented by testosterone/DHT binding to AR and stimulating the transcription of AR target genes (Davey and Grossmann 2016). Given its critical role in normal prostate cells, prostate carcinogenesis and subsequent phases of disease progression are regulated positively in the presence of androgens. After androgen-ablation therapy, the standard treatment for PCa, tumor size decreases because of the reduced viability of androgen-dependent cells. However, this course of treatment often results in the recurrence of androgen-independent tumors, which originate from more aggressive and metastatic cells of the previous population (Nelson et al., 2003; Abate-Shen et al., 2008).

Variations in AR signaling have also been reported in the settings of chronic inflammation (Debelec-Butuner et al., 2012). The presence of androgens at normal physiological levels plays a vital role in maintaining the homeostasis between pro-oxidant and antioxidant components as well as between cell death and proliferation of normal tissues in the prostate. In castration-induced androgen deprivation, the normal androgenic state is disrupted, and oxidative stress is induced by increasing NOX-dependent ROS anabolism, which leads to decreased antioxidant enzymatic levels (Miyata et al., 2017).

*NKX3.1* is an androgen-regulated homeobox gene (Bieberich et al., 1996) with a basal expression in prostate cells. It is upregulated by AR-mediated transcription (Korkmaz et al., 2000). The function of NKX3.1 is necessary for normal prostate development, and its loss is associated with PCa development. Expression of NKX3.1 is downregulated in inflammatory atrophy and preinvasive PCa (Bowen et al., 2000; Debelec-Butuner et al., 2012). Besides its tumor-suppressive function, NKX3.1 also protects cells from oxidative damage (Ouyang et al., 2005; Debelec-Butuner et al., 2019). In addition, NKX3.1 interacts with DNA topoisomerase I through its homeodomain and increases the ability of enzymes to bind to DNA, thereby enhancing DDR in the ATM-mediated damage repair pathway (Bowen et al., 2007). Proinflammatory cytokines such as TNF-α and IL1-β trigger the loss of NKX3.1 and AR by altering protein stability (Markowski et al., 2008; Debelec-Butuner et al., 2012), thereby inducing ubiquitination and proteasomal degradation.

The mediator of DNA damage checkpoint protein 1 (MDC1), which functions in DDR was identified as a coactivator of AR raising new questions on the contribution of AR in DDR (Wang et al., 2015). The critical role of AR as a regulator of DDR genes has been previously reported, leading to the combination of DDR inhibitors and AR deprivation therapy as a new treatment strategy for aggressive prostate cancer (Karanika et al., 2015; Rao et al., 2022; Unlu and Kim 2022).

In this study, we aimed to reveal the effects of AR and NKX3.1 loss on ROS-induced DNA damage level and DDR mechanisms under oxidant and inflammatory conditions of tumor cells. The normal prostate epithelial cell line RWPE1, androgen-responsive PCa cell line LNCaP, and castration-resistant PCa cell line LNCaP-104r2 were used to study the cellular levels of ROS and ROS-induced DSB damage. Apart from the repair mediators, in observing the relative effects of expressional changes in AR and NKX3.1, we performed AR and/or NKX3.1 knockdown, then cells were treated with either oxidants or inflammatory microenvironment exposures.

## 2. Materials and methods

### 2.1. Cell culture

LNCaP, U937, and RWPE-1 cells were obtained from the American Type Culture Collection (Manassas, VA), and LNCaP-104r2 cells were provided by John M. Kokontis (Chuu et al., 2011). LNCaP and U937 cells were propagated using RPMI 1640 (Gibco-Invitrogen, US) supplemented with 10% FBS, L-glutamine (2 mM), penicillin (100 U/mL), and streptomycin (100 mg/mL), whereas RWPE-1 cells were propagated in keratinocyte growth medium supplemented with bovine pituitary extract and 5 mM EGF at 37 °C with 5% CO_2_. The LNCaP-104r2 cells were propagated using RPMI 1640 supplemented with 3% charcoal-treated FBS, L-glutamine (2 mM), penicillin (100 U/mL), and streptomycin (100 mg/mL) at 37 °C with 5% CO_2_.

### 2.2. Macrophage differentiation and CM collection

Macrophage differentiation and cytokine production were performed by using U937 cells according to the previous protocols (Debelec-Butuner et al., 2012).

### 2.3. Measurement of TNFα concentration in CM

TNF-α levels in CM were analyzed using an ELISA according to the manufacturer’s instructions (Invitrogen, US). TNFα was selected as a measure of CM concentration based on previous studies (Debelec-Butuner et al., 2012). The concentration of TNFα in CM treatments was adjusted by diluting the CM with a regular medium before treatment to LNCaP cells.

### 2.4. Treatments

Conditioned media (CM) treatments containing 62, 125, and 250 pg/mL of TNF-α were performed for 24 h. For chronic inflammatory conditions, CM treatments were performed for 2 weeks for chronic inflammatory conditions at CM concentrations containing 50 and 100 pg/mL of TNF-α. TNFα concentrations were adjusted by diluting the CM using the RPMI 1640 medium as described previously (Debelec-Butuner et al., 2012). To compare the effects of cytokine exposure and oxidative stress, we treated the cells in the presence of 50, 100, or 200 μM H_2_O_2_ for 2 weeks under a chronic oxidative condition. Treatments with N-acetyl-L-cysteine (L-NAC; 10 mM) and R1881 (10 nM) (Debelec-Butuner et al., 2012; Debelec-Butuner et al., 2015) were performed for 1 h prior to CM treatments and maintained until harvest.

### 2.5. Transfections

The NKX3.1 overexpression was performed according to our previous publication (Debelec-Butuner et al., 2012). Transfections were performed on 4 × 10^5^ cells using the Xtreme reagent (Roche, Germany) for 24 h. siAR transfections were performed in accordance with the supplier’s recommendation (Dharmacon, US). Briefly, 4 × 10^5^ cells were cultured in a 6-cm plate, and the medium was changed (without antibiotics) after 48 h. A transfection mix was prepared by adding 6 μL of Dharmafect II (tube 1) and 200 pmol of *siAR* or scrambled siRNA (tube 2) into 94 μL of transfection medium (without antibiotics and serum). After incubation for 5 min at room temperature (RT), the tubes were mixed and incubated further for 15 min at RT and then dispensed onto the cells dropwise. The transfected cells were incubated for an additional 24 h before harvesting.

### 2.6. DCFH-DA intracellular ROS measurement assay

LNCaP cells (8 × 10^3^) were cultured in 96-well plates, and transfections were carried out on the following day. Two days later, the cells were incubated with 10 μM DCFH-DA (2′ 7′-dichlorodihydrofluorescein diacetate, Molecular Probes, US) for 30 min at 37 °C. After treatments, the cells were gently washed using a phenol-red-free medium. Finally, the fluorescence intensity was measured every 20 min at 37 °C for up to 3 h using a Fluoroskan microplate reader (Thermo Fisher Scientific, US).

### 2.7. Foci analysis using the Aklides Cell damage platform

Aklides Cell Damage (Medipan, Dahlewitz, Germany) is an automated immunofluorescence-based gH2AX foci detection system that involves high-resolution semiconfocal immunofluorescence microscopy and sophisticated image analysis software (Reddig et al., 2015; Reddig et al., 2021). Experimentally, the cells were cultured on glass coverslips in 6-well plates until reached the desired confluency. All cells were washed once with PBS and fixed by incubation with methanol (99.5%) at −20 °C for 30 min. Cells were permeabilized with 0.2% Triton X-100 in PBS for 5 min on a shaker and blocked with 1% BSA in PBS for 5 min. The cells were stained by incubation with anti-gH2AX antibody (1/250 dilution in 1% BSA in PBS) in a humidified chamber for 1 h, followed by incubation with a 1:1000 dilution of Alexa Fluor 488-conjugated antirabbit at RT for 20 min. The stained coverslips were placed onto Aklides Cell damage slides. The parameters (nucleus diameter, nucleus height/width ratio, foci diameter, foci intensity, and foci convexity) of the software were adjusted to the previously optimized values based on the cell type (LNCaP). In addition, foci were defined in five focal planes and counted. The results indicating foci number/intensity for each cell were given by the system software, which was used for further analysis, and the data were analyzed. The average γH2AX^S139^ foci number for each cell and the percentage of cells with the indicated number of foci (at least 100 cells for each sample) were plotted.

### 2.8. Protein extraction and Western blotting

Protein extraction, SDS-PAGE, and Western blots were performed according to our previous paper under standard conditions with 50 μg of protein lysate per lane (Debelec-Butuner et al., 2012). The following antibodies were purchased and used according to the manufacturer’s recommendations: AR (Cat. no: 06680, Millipore, US); p21 (Cat. no: sc-817), NRF2 (Cat. no: sc-722), and ATM (Cat. no: sc-23921, Santa Cruz Biotech., US); γH2AX^S139^ (Cat. no: ab11174), p-Nrf2^S40^ (Cat. no: ab76026), ac-p53(K382) (Cat. no: ab75754), and pATM^S1981^ (Cat. no: ab81292, Abcam, UK); GAPDH (Cat. no: AM4300, Ambion, UK); β-actin (Cat. no: A3854) and SIRT1 (Cat. no: S5447, Sigma, US); Caspase-3 (Cat. no: AF-605-NA, R&D) and β-tubulin (Cat. no: G098, ABM); HRP-conjugated antimouse and antirabbit (Cat. no: NA931V and NA934V, Amersham, UK). The NKX3.1 custom antibody was produced in Prof. Dr. F. Saatcioglu (University of Oslo) laboratory, used in previous literature (Korkmaz et al., 2004), and provided us as a gift.

### 2.9. Statistics

Statistical analyses were performed with Prism 8.0 (GraphPad). Data sets were analyzed with parametric 2-tailed Student’s t-tests. p values for the pairs < 0.05 were considered significant and shown as follows * p < 0.05, ** p < 0.01 and *** p < 0.001. Data are presented as mean values ± SEM of triplicate treatments (n = 3).

## 3. Results

### 3.1. Loss of AR function elicits increased intracellular ROS and DNA damage

**Figure 1:**
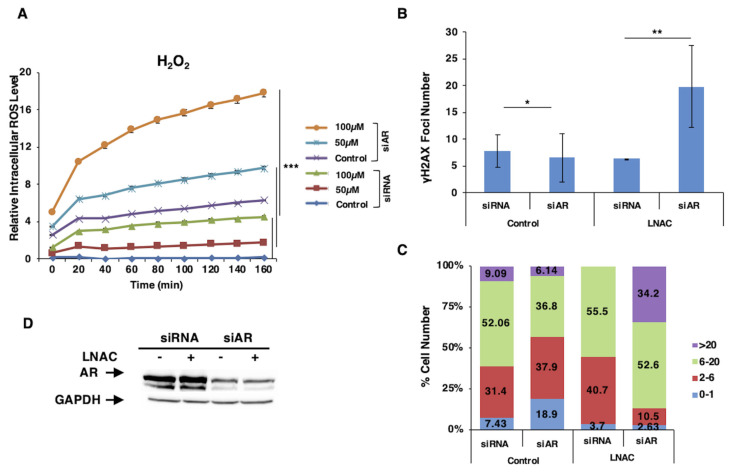
The effect of AR silencing on intracellular ROS level and oxidative DNA damage recognition in LNCaP cells. A. AR silencing for 24 h was followed by H_2_O_2_ (50 and 100 μM) treatment, and the relative fluorescence level was measured every 20 min for 3 h using a fluorimeter by the DCFH method. Time-dependent changes in intracellular ROS are presented. Data are presented as mean values ± SEM of triplicate treatments (n = 3). B, C, D. Following 24 h of AR silencing and LNAC (10 mM) treatment (1 h prior to transfection), the number of γH2AX^S139^ foci was analyzed and counted using the Aklides Cell damage system and B. average number of foci per cell (p values for the pairs were *p < 0.05, **p < 0.01 and ***p < 0.001) C. % of cells carrying the indicated number of foci (0–1, 2–6, 6–20, and >20 foci) D. Western blot analysis confirming AR silencing were presented. scr: control siRNA; siAR: androgen receptor siRNA; LNAC: N-Acetyl-L-cysteine.

In understanding the importance of AR upon intracellular ROS levels, DNA damage, and repair activation, AR knockdown was performed by silencing LNCaP cells. Cells were transfected with either control siRNA or siAR and then treated with 50 and 100 μM H_2_O_2_. Intracellular ROS level increased in a concentration-dependent manner, and this increase was significantly augmented when the AR was silenced. Furthermore, a remarkable increase in ROS level with AR silencing alone in the absence of oxidant conditions indicated that AR signaling plays a key role in the regulation of intracellular ROS caused by metabolic oxidative respiration. Considering that the increase in ROS level after H_2_O_2_ exposure is higher in AR-silenced cells than in AR-expressing ones, AR might play an important role in the antioxidant response mechanism in prostate cells (Figure 1A). Considering that the increased intracellular ROS level also induces DNA damage when oxidative stress is relatively high because of the impaired metabolic control-mediated stress tolerance and antioxidant response, we aimed to investigate the DNA damage level upon the lack of AR-mediated oxidative stress control. Therefore, the number of γH2AX foci was analyzed in the absence and presence of AR silencing and antioxidant N-Acetyl-L-cysteine (LNAC) in LNCaP cells by using the Aklides Cell damage system. The loss of AR signaling could slightly decrease the average number of γH2AX foci, which was reversed by LNAC treatment (Figure 1B). In addition, analyzing the data as the percentage of cells with a certain number of foci showed that AR silencing decreased the percentage of cells with more DNA damage foci but increased in the presence of antioxidant LNAC with AR silencing (Figure 1C). Western blot analysis was performed to confirm decreased AR protein level upon silencing in LNCaP cells (Figure 1D).

**Figure 2:**
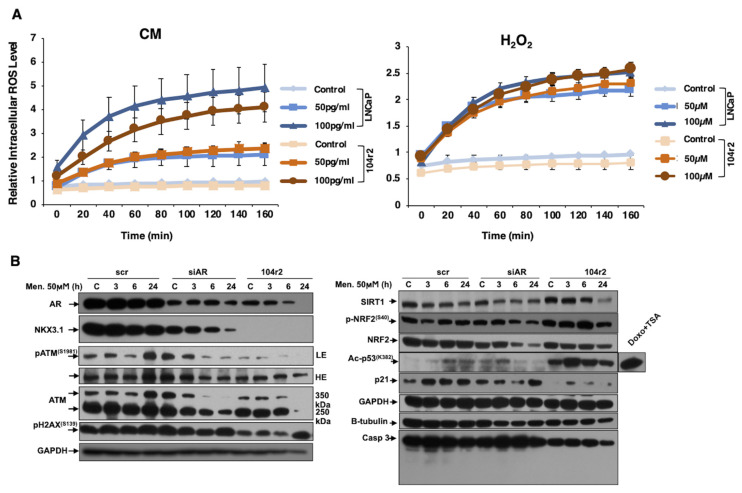
Comparison of the functional loss of AR on intracellular ROS level and DNA damage recognition mechanism in androgen-responsive LNCaP and castration-resistant LNCaP 104r2 cells. A. Following CM (inflammatory medium containing 50 and 100 pg/mL of TNF-α) and H_2_O_2_ (50 and 100 μM) treatments, the relative fluorescence level was measured every 20 min for 3 h using a fluorimeter by the DCFH method. Time-dependent changes in intracellular ROS levels are presented. Data are presented as mean values ± SEM of triplicate treatments (n = 3). B. Transfection of LNCaP cells with control siRNA (scr) or siAR for 24 h was followed by treatment of transfected LNCaP cells and LNCaP 104r2 cells with 50 μM menadione for 3, 6, and 24 h. Protein levels of AR and the tumor suppressor protein NKX3.1, which is transcriptionally controlled by AR, were analyzed. In investigating the DDR mechanism, pATM^S1981^, ATM, and γH2AX^S139^ levels were examined. GAPDH is presented as the loading control. Protein levels of SIRT1, Nrf2, pNrf2^S40^, ac-p53^K382^, p21, and Caspase-3 were examined. GAPDH and β-tubulin are presented as loading control. Cotreatment with doxorubicin and trichostatin A (TSA) is presented as a positive control of p53 acetylation. scr: control siRNA; siAR: androgen receptor siRNA.

Furthermore, the effect of the abrogated AR signaling on intracellular ROS levels was investigated in androgen-responsive LNCaP and castration-resistant LNCaP 104r2 (androgen-independent) cells. Measurement of ROS levels showed no significant difference in basal level and oxidant conditions upon exposure to an inflammatory microenvironment medium and H_2_O_2_ (Figure 2A). In investigating the contribution of the AR pathway to DDR under oxidative conditions, menadione (as a more stable oxidant) treatment was performed for 3, 6, and 24 h (0.05 mM) in LNCaP 104r2 and LNCaP cells following AR silencing. Alterations in protein expression related to DDR were examined. AR expression was lower in 104r2 cells than in LNCaPs, whereas the AR-regulated basal expression level of NKX3.1 was slightly decreased upon AR silencing in LNCaPs, and it was not detectable in 104r2 cells because of diminished AR signaling. Moreover, time-dependent increases of pATM^S1981^ and γH2AX^S139^ were enhanced because of AR silencing only at early time points; however, they were at lower levels in 104r2 cells compared with those in LNCaPs, indicating the low level of basal DNA damage in 104r2 cells. Accordingly, basal and menadione-induced levels of pNRF2^S40^ and SIRT1 in 104r2 cells were higher than those in LNCaPs, indicating that the enhanced antioxidant response and DDR activation occur simultaneously. The expression level of pNRF2^S40^, NRF2, and SIRT1 decreased in a time-dependent manner when AR was silenced in LNCaP cells. In addition, induced p53^K382^ acetylation in line with the time-dependent increase of DNA damage level was lower after AR silencing but significantly higher in 104r2 cells leading to enhanced p53 stabilization. p21 activation observed in AR-positive cells remained unchanged in AR-silenced cells, whereas marginal p21 activation was detected in 104r2 cells with lower basal expression levels of p21 and caspase 3, which are *bona fide* regulators of cell cycle and apoptosis (Figure 2B).

**Figure 3:**
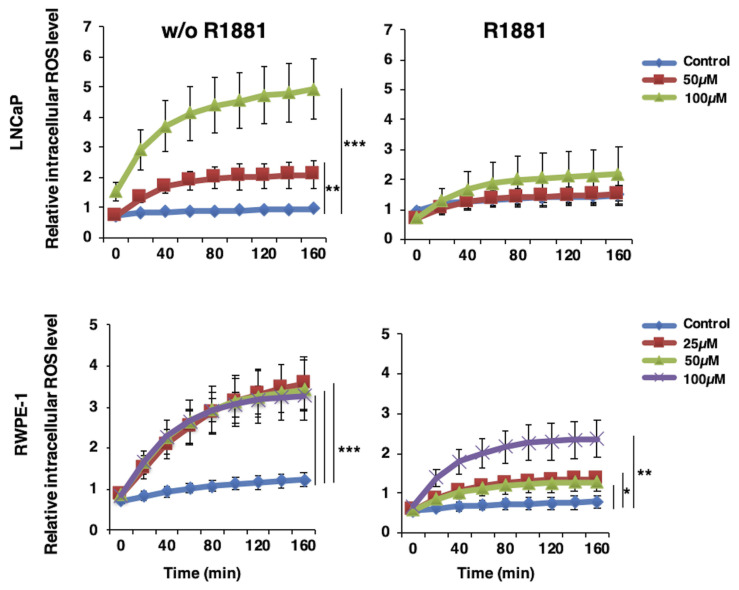
Effect of synthetic androgen administration on intracellular ROS levels in LNCaP and RWPE-1. Following H_2_O_2_ (25, 50, and 100 μM) and R1881 (10nM) treatments, relative fluorescence values were measured using a fluorimeter by the DCFH method. Time-dependent changes in intracellular ROS levels are presented. Data are presented as mean values ± SEM of triplicate treatments (n = 3). p values for the pairs were *p < 0.05, **p < 0.01 and ***p < 0.001.

The effect of AR activation triggered by androgen treatment on the intracellular ROS level was investigated in LNCaP cells and normal prostate epithelial cell line RWPE-1. 10^4^ cells were first treated with 10 nM R1881, a synthetic androgen, for 24 h and then treated with 25, 50, and 100 μM H_2_O_2_ in the presence or absence of R1881. The ROS level was analyzed for 3 h by using the DCFH assay. Androgen treatment could alleviate intracellular ROS induced by H_2_O_2_ (Figure 3), confirming the role of androgens likely via AR in oxidative stress regulation.

Consequently, AR signaling has a suppressive role in the regulation of intracellular ROS in androgen-responsive prostate cancer cells but not in castration-resistant cells. Further, AR silencing resulted in decreased DNA damage recognition, which was reversed by antioxidant conditions. Besides, lower DNA damage recognition occurred in castration-resistant cells with higher tolerance to oxidant conditions.

### 3.2. AR-regulated NKX3.1 reduces DNA damage under oxidant and inflammatory conditions

**Figure 4:**
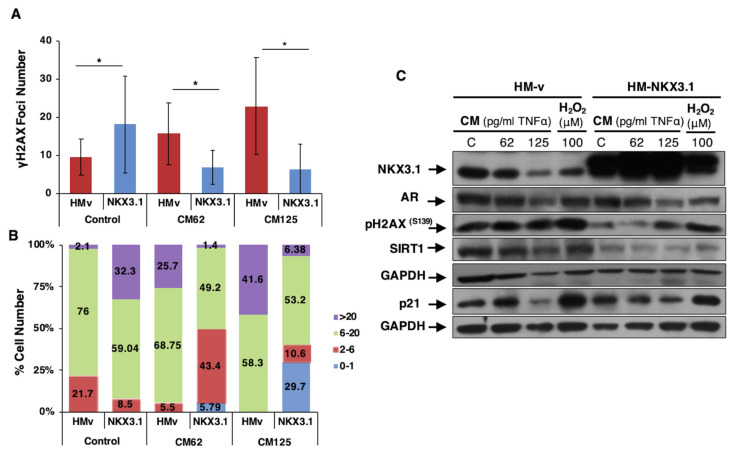
Effect of NKX3.1 expression on oxidative stress-mediated DNA damage and damage recognition mechanism in LNCaP cells. A. LNCaP cells were transfected with HM-vector and HM-NKX3.1 and 24 h later, CM treatment (inflammatory medium containing 62 and 125 pg/mL of TNF-α) was performed for 24 h. The number of γH2AX^S139^ foci was analyzed and counted using the Aklides Cell damage system and the average number of foci per cell (P values for the pairs were *P < 0.05 and **P < 0.01). B. % of cells carrying the indicated number of foci (0–1, 2–6, 6–20, and >20 foci) were presented. C. Treatments (CM containing 62 and 125 pg/mL of TNF-α or H_2_O_2_ 100 μM) were performed for 24 h following the transfection of the HM-vector and HM-NKX3.1 24 h prior. The expression level of the players’ DNA damage recognition and repair activation mechanism was investigated. GAPDH served as the loading control.

The role of the diminished antioxidant response mechanism on the accumulation of oxidative DNA damage during tumorigenesis led us to examine the effects of NKX3.1, of which cellular levels decreased in the inflammatory microenvironment because of androgen ablation therapy as a member of the androgen signaling pathway. Considering that the loss of antioxidant response to oxidants in the inflammatory microenvironment in LNCaP cells was demonstrated in our previous publication (Debelec-Butuner et al., 2015), the role of NKX3.1 on DNA damage formation and damage response activation was investigated. NKX3.1 expression was ectopically increased in LNCaP cells, and CM treatment containing 62 and 125 pg/mL of TNF-α was performed for 24 h to examine the effect of NKX3.1 on γH2AX^S139^ foci number. Interestingly, the more CM concentration increased, the more the average level of γH2AX^S139^ (Figure 4A) and the percentage of cells with a relatively high number of foci (Figure 4B) increased. Although NKX3.1 expression could increase the foci number in normal cellular conditions, probably because of enhanced DNA damage recognition in the presence of NKX3.1, DNA damage was suppressed in the inflammatory microenvironment (Figures 4A and B). Furthermore, to examine DDR to oxidants in the presence of NKX3.1, LNCaP cells were transfected with HM-vector and HM-NKX3.1 and then treated with CM containing 62 and 125 pg/mL of TNF-α and 100 μM H_2_O_2_ for 24 h. Reduced γH2AX^S139^ levels were observed in cells with enhanced NKX3.1 expression. In addition, SIRT1 levels were suppressed in highly NKX3.1-expressing cells, indicating that NKX3.1 reduces DNA damage, thereby decreasing the level of SIRT1 as the modulator of DDR without metabolic activation of prostate cells. Moreover, altered p21 expression under inflammatory and oxidant conditions was correlated to NKX3.1 level in natively NKX3.1-expressing cells. Furthermore, reduced DNA damage by enhanced NKX3.1 expression could stabilize p21 levels (Figure 4C). These results showed that increased DNA damage in inflammatory and oxidant cellular conditions was alleviated by NKX3.1 expression showing its loss in DDR mechanism.

### 3.3. Loss of AR signaling in chronic inflammation but not in oxidative stress increases the number of cells with heavy DNA damage

**Figure 5:**
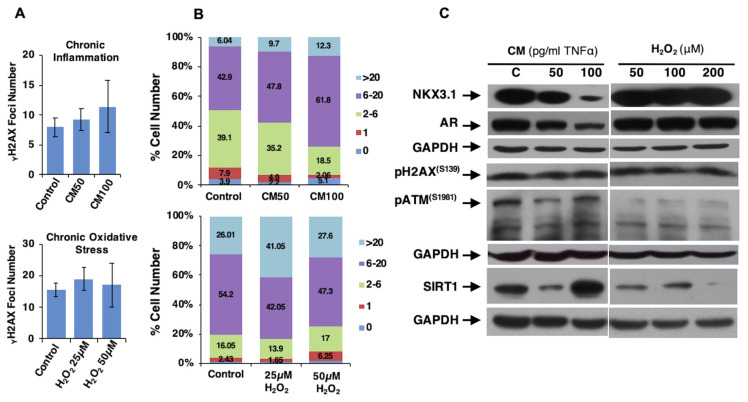
Effect of chronic inflammatory or oxidative microenvironment on the DNA damage recognition mechanism. LNCaP cells were treated with either CM (inflammatory medium containing 50 and 100 pg/mL of TNF-α) or H_2_O_2_ (25, 50, 100, and 200 μM) for 2 weeks. A. The number of γH2AX^S139^ foci was analyzed and counted using the Aklides Cell damage system and the average number of foci per cell. B. % of cells carrying the indicated number of foci (0–1, 2–6, 6–20, and >20 foci) were presented. C. The expression level of the players’ DNA damage recognition and repair activation mechanism was investigated. GAPDH served as the loading control.

Considering that molecular changes during chronic inflammation and oxidative stress are associated with tumorigenesis and metastasis (Debelec-Butuner et al., 2012; Debelec-Butuner et al., 2014), the cellular effects of AR and NKX3.1 loss in the chronic inflammatory microenvironment on DNA damage level and repair activation in comparison with chronic oxidant conditions were examined. Relatively low doses of H_2_O_2_ (25, 50, and 100 μM) or CM (containing 50 and 100 pg/mL of TNF-α) were applied to cells for 2 weeks to mimic the cellular microenvironment of chronic inflammatory and oxidative stress conditions in cell culture. The γH2AX^S139^ foci quantitation showed a dose-dependent marginal increase in the average γH2AX^S139^ foci number but a significant increase in the percentage of cells carrying a higher number of foci in chronic inflammation. Long-term treatment of H_2_O_2_ only increased DNA damage at a low dose, probably because of the enhanced activation of antioxidant response upon high doses (Figures 5A and 5B). Expressional alterations indicated proteasomal degradation of AR and NKX3.1 upon CM treatment but not in relatively mild chronic oxidative stress conditions. Although the γH2AX^S139^ protein level remained unchanged, which was correlated with the slight change in the quantification of γH2AX^S139^ foci, pATM^S1981^ was alleviated upon treatment with H_2_O_2_ and a lower dose of CM. Increased SIRT1 expression was observed in chronic inflammatory conditions when only accompanied by significant loss of AR and NKX3.1 (Figure 5C). As a result, abrogated AR signaling, including NKX3.1 loss, increased the number of cells with heavy DNA damage and SIRT1 activation, particularly in chronic inflammatory conditions.

## 4. Discussion

Prostate cancer is the most diagnosed cancer and the second leading cause of cancer-related mortality among men in Western countries. AR is known to play a crucial role in the growth and progression of PCa. Considering that PCa is androgen dependent, particularly at the early stage of cancer, the inhibition of AR signaling via androgen deprivation therapy (ADT) is used as the first-line treatment (Koivisto et al., 1998; Miller et al., 2021). In this study, we aimed to understand the effects of the loss of AR signaling upon ADT on DNA damage formation and repair activation mechanisms under oxidant and inflammatory conditions of prostate tumor microenvironment.

Increased intracellular ROS after AR silencing confirms the significant role of AR signaling in oxidative stress regulation in the tumor microenvironment during PCa progression. Furthermore, the remarkably increased ROS level in AR silencing without oxidant treatment indicates that AR signaling is endogenously necessary to regulate produced ROS levels (Figure 1).

H_2_O_2_ and CM exposures, which were used to mimic the oxidant and inflammatory tumor microenvironment, respectively, in LNCaP and LNCaP-104r2 cells revealed no significant difference in intracellular ROS levels between androgen-responsive and castration-resistant cells. However, expressional changes of the members of the DNA damage repair activation mechanism upon oxidant conditions confirmed the significant role of AR and AR-regulated NKX3.1 in DNA damage recognition and repair activation. The increase of γH2AX^S139^ and pATM^S1981^ protein levels because of AR silencing in the absence of oxidants may be compatible with the enhanced intracellular ROS and DNA damage levels quantitated by AKLIDES. Based on the results showing higher expression levels of pNRF2^(S40)^ and NRF2 in 104r2 cells compared to LNCaPs, castration-resistant PCa cells, which have almost the same intracellular ROS levels under oxidant conditions, show higher activation of antioxidant responses. These cells with better antioxidant capacity were determined to have lower DNA damage levels, as shown by γH2AX^S139^ and pATM^S1981^ phosphorylation. In addition, enhanced levels of SIRT1 and ac-p53^(K382)^ in 104r2 cells indicate that SIRT1 plays an important role in the remarkable activation of DDR in these cells even under the same oxidant conditions, leading to enhanced repair activation and prevented cell death (Figure 2). Moreover, decreased ROS levels in PCa and normal prostate epithelial cells in the presence of R1881 confirmed the functional role of androgens in oxidative stress regulation in prostate cell metabolism (Figure 3).

The proteasomal degradation-mediated loss of NKX3.1 in the presence of inflammatory cytokines can disturb its tumor-suppressive function during PCa initiation (Markowski et al., 2008). However, in NKX3.1-expressing cancer cases, the expressional loss of NKX3.1 after androgen deprivation therapy also results in functional loss, leading to deregulated activation of antioxidant response (Debelec-Butuner et al., 2015) and ATM-mediated DDR mechanisms (Bowen et al., 2013). The ectopic expression of NKX3.1 under inflammatory conditions resulted in lower levels of γH2AX^S139^ accompanied by a SIRT1 decrease and p21 stabilization, which indicate that the regulatory function of NKX3.1 in the antioxidant response mechanism is predominant, thereby alleviating ROS-induced DNA damage (Figure 4).

Results showing the degradation of AR and NKX3.1 under chronic inflammatory conditions but stabilization under chronic oxidant conditions allowed us to perceive the role of AR signaling-mediated DNA repair activation in prostate cells. Their loss in inflammatory conditions resulted in higher DNA damage levels with activated SIRT1 compared with H_2_O_2_ oxidant conditions (Figure 5), which is correlated with the results of NKX3.1 overexpression.

In addition, AR and NKX3.1 led to decreased DNA damage levels through their oxidative stress regulatory functions. Moreover, NKX3.1 along with the ATM complex is involved in DNA damage recognition, resulting in increased γH2AX^S139^ phosphorylation, indicating the recognition of the damage by the response mechanism. This phosphorylation is necessary to trigger DSB repair, and the effects of the NKX3.1 protein on γH2AX phosphorylation levels have been demonstrated in our studies. This result also correlates with a previous study reporting the positive role of NKX3.1 in DNA repair activity by influencing the recruitment of homology-directed DNA repair proteins (Bowen et al., 2015). Although the effect of NKX3.1 on the recognition of DNA damage might be more pronounced in relatively lower oxidative stress conditions, its function on oxidative stress regulation might be prominent under highly damaging cellular conditions (Takeda et al., 1999; Teramoto et al., 1999; Saito et al., 2006; Reuter et al., 2010).

The balance between the antioxidant response and DDR activation should be carefully investigated, particularly under the inflammatory condition of the tumor microenvironment. During the loss of AR and AR-regulated NKX3.1 because of either an inflammatory microenvironment or ADT, ROS-scavenging antioxidant levels are decreased; ATM-mediated DDR is not activated; SIRT1-mediated metabolic control is suppressed, and DNA damage is ultimately accumulated, leading to genomic instability in prostate cells. Considering that the AR signaling pathway affects the formation and recognition of DNA damage, the detection and evaluation of the cellular results of antiandrogens on DNA damage formation and repair activation mechanisms must be further investigated.

Based on the literature and current results, oxidative stress and its cellular effects contribute to not only PCa initiation and progression but also the transition to a castration-resistant state (Tam et al., 2003; De Marzo et al., 2007; Klein and Silverman 2008; Khalili et al., 2010). Therapy-based alterations in oxidative stress regulation and DDR mechanisms should be investigated to achieve optimized therapy strategies. Further, crosstalk between the DNA damage repair and androgen receptor signaling through regulating genes involved in the homolog recombination and nonhomolog end joining pathway was reported recently (Jividen et al., 2018; Zhang et al., 2020), confirming the emerging role of DDR-based therapy strategies (Burdak-Rothkamm et al., 2020; Zhang et al., 2020). Our results also indicate that castration-resistant PCa cells have a higher antioxidant response, causing them to tolerate oxidant conditions and to be more resistant to oxidative DNA damage, thereby resulting in the therapy resistance of these cells. Furthermore, AR signaling is vital for the regulation of oxidative stress and DDR mechanisms. Thus, the loss of AR signaling upon antiandrogen therapy should be reconsidered. Such knowledge would permit opening the door for the pursuit of new general therapeutic strategies to treat PCa.

## References

[b1-turkjbiol-47-5-325] Abate-Shen C Shen MM Gelmann E 2008 Integrating differentiation and cancer: the Nkx3.1 homeobox gene in prostate organogenesis and carcinogenesis Differentiation 76 717 727 10.1111/j.1432-0436.2008.00292.x 18557759 PMC3683569

[b2-turkjbiol-47-5-325] Bieberich CJ Fujita K He WW Jay G 1996 Prostate-specific and androgen-dependent expression of a novel homeobox gene The Journal of Biological Chemistry 271 31779 31782 10.1074/jbc.271.50.31779 8943214

[b3-turkjbiol-47-5-325] Bowen C Bubendorf L Voeller HJ Slack R Willi N 2000 Loss of NKX3.1 expression in human prostate cancers correlates with tumor progression Cancer Research 60 6111 6115 11085535

[b4-turkjbiol-47-5-325] Bowen C Ju JH Lee JH Paull TT Gelmann EP 2013 Functional activation of ATM by the prostate cancer suppressor NKX3.1 Cell Reports 4 516 529 10.1016/j.celrep.2013.06.039 23890999 PMC3838670

[b5-turkjbiol-47-5-325] Bowen C Stuart A Ju JH Tuan J Blonder J 2007 NKX3.1 homeodomain protein binds to topoisomerase I and enhances its activity Cancer Research 67 455 464 10.1016/j.celrep.2013.06.039 17234752

[b6-turkjbiol-47-5-325] Bowen C Zheng T Gelmann EP 2015 NKX3.1 Suppresses TMPRSS2-ERG Gene Rearrangement and Mediates Repair of Androgen Receptor-Induced DNA Damage Cancer Research 75 2686 2698 10.1158/0008-5472.CAN-14-3387 25977336 PMC4511965

[b7-turkjbiol-47-5-325] Burdak-Rothkamm S Mansour WY Rothkamm K 2020 DNA Damage Repair Deficiency in Prostate Cancer Trends in Cancer 6 974 984 10.1016/j.trecan.2020.05.011 32517958

[b8-turkjbiol-47-5-325] Burma S Chen BP Murphy M Kurimasa A Chen DJ 2001 ATM phosphorylates histone H2AX in response to DNA double-strand breaks The Journal of Biological Chemistry 276 42462 42467 10.1074/jbc.C100466200 11571274

[b9-turkjbiol-47-5-325] Chuu CP Kokontis JM Hiipakka RA Fukuchi J Lin HP 2011 Androgen suppresses proliferation of castration-resistant LNCaP 104.R2 prostate cancer cells through androgen receptor, Skp2, and c-Myc Cancer Science 102 2022 2028 10.1111/j.1349-7006.2011.02043.x 21781227 PMC3200457

[b10-turkjbiol-47-5-325] Davey RA Grossmann M 2016 Androgen Receptor Structure, Function and Biology: From Bench to Bedside The Clinical Biochemist Reviews 37 3 15 27057074 PMC4810760

[b11-turkjbiol-47-5-325] De Marzo AM Platz EA Sutcliffe S Xu J Gronberg H 2007 Inflammation in prostate carcinogenesis Nature Review Cancer 7 256 269 10.1038/nrc2090 17384581 PMC3552388

[b12-turkjbiol-47-5-325] Debelec-Butuner B Alapinar C Ertunc N Gonen-Korkmaz C Yorukoglu K 2014 TNFalpha-mediated loss of beta-catenin/E-cadherin association and subsequent increase in cell migration is partially restored by NKX3.1 expression in prostate cells PloS One 9 e109868 10.1371/journal.pone.0109868 25360740 PMC4215977

[b13-turkjbiol-47-5-325] Debelec-Butuner B Alapinar C Varisli L Erbaykent-Tepedelen B Hamid SM 2012 Inflammation-mediated abrogation of androgen signaling: An in vitro model of prostate cell inflammation Molecular Carcinogenesis 53 85 97 10.1002/mc.21948 22911881

[b14-turkjbiol-47-5-325] Debelec-Butuner B Bostanci A Ozcan F Singin O Karamil S 2019 Oxidative DNA Damage-Mediated Genomic Heterogeneity Is Regulated by NKX3.1 in Prostate Cancer Cancer Investigation 37 113 126 10.1080/07357907.2019.1576192 30836777

[b15-turkjbiol-47-5-325] Debelec-Butuner B Ertunc N Korkmaz KS 2015 Inflammation contributes to NKX3.1 loss and augments DNA damage but does not alter the DNA damage response via increased SIRT1 expression Journal of Inflammation 12 12 10.1186/s12950-015-0057-4 25705129 PMC4336697

[b16-turkjbiol-47-5-325] Ehsani M David FO Baniahmad A 2021 Androgen Receptor-Dependent Mechanisms Mediating Drug Resistance in Prostate Cancer Cancers 13 1534 10.3390/cancers13071534 33810413 PMC8037957

[b17-turkjbiol-47-5-325] Finkel T 2003 Oxidant signals and oxidative stress Current Opinion in Cell Biology 15 247 254 10.1016/s0955-0674(03)00002-4 12648682

[b18-turkjbiol-47-5-325] Jividen K Kedzierska KZ Yang CS Szlachta K Ratan A 2018 Genomic analysis of DNA repair genes and androgen signaling in prostate cancer BMC Cancer 18 960 10.1186/s12885-018-4848-x 30305041 PMC6180441

[b19-turkjbiol-47-5-325] Karanika S Karantanos T Li L Corn PG Thompson TC 2015 DNA damage response and prostate cancer: defects, regulation and therapeutic implications Oncogene 34 2815 2822 10.1038/onc.2014.238 25132269 PMC4333141

[b20-turkjbiol-47-5-325] Khalili M Mutton LN Gurel B Hicks JL De Marzo AM 2010 Loss of Nkx3.1 expression in bacterial prostatitis: a potential link between inflammation and neoplasia The American Journal of Pathology 176 2259 2268 10.2353/ajpath.2010.080747 20363913 PMC2861091

[b21-turkjbiol-47-5-325] Khandrika L Kumar B Koul S Maroni P Koul HK 2009 Oxidative stress in prostate cancer Cancer Letters 282 125 136 10.1016/j.canlet.2008.12.011 19185987 PMC2789743

[b22-turkjbiol-47-5-325] Klein EA Silverman R 2008 Inflammation, infection, and prostate cancer Current Opinion in Urology 18 315 319 10.1097/MOU.0b013e3282f9b3b7 18382242

[b23-turkjbiol-47-5-325] Koivisto P Kolmer M Visakorpi T Kallioniemi OP 1998 Androgen receptor gene and hormonal therapy failure of prostate cancer The American Journal of Pathology 152 1 9 9422516 PMC1858130

[b24-turkjbiol-47-5-325] Korkmaz CG Korkmaz KS Manola J Xi Z Risberg B 2004 Analysis of androgen regulated homeobox gene NKX3.1 during prostate carcinogenesis The Journal of Urology 172 1134 1139 10.1097/01.ju.0000136526.78535.b8 15311057

[b25-turkjbiol-47-5-325] Korkmaz KS Korkmaz CG Ragnhildstveit E Kizildag S Pretlow TG 2000 Full-length cDNA sequence and genomic organization of human NKX3A - alternative forms and regulation by both androgens and estrogens Gene 260 25 36 10.1016/s0378-1119(00)00453-4 11137288

[b26-turkjbiol-47-5-325] Liu B Chen Y St Clair DK 2008 ROS and p53: a versatile partnership Free Radical Biology Medicine 44 1529 1535 10.1016/j.freeradbiomed.2008.01.011 18275858 PMC2359898

[b27-turkjbiol-47-5-325] Markowski MC Bowen C Gelmann EP 2008 Inflammatory cytokines induce phosphorylation and ubiquitination of prostate suppressor protein NKX3.1 Cancer Research 68 6896 6901 10.1158/0008-5472.CAN-08-0578 18757402 PMC2586101

[b28-turkjbiol-47-5-325] Miller KD Ortiz AP Pinheiro PS Bandi P Minihan A 2021 Cancer statistics for the US Hispanic/Latino population, 2021 CA: A Cancer Journal for Clinicians 71 466 487 10.3322/caac.21695 34545941

[b29-turkjbiol-47-5-325] Miyata Y Matsuo T Sagara Y Ohba K Ohyama K 2017 A Mini-Review of Reactive Oxygen Species in Urological Cancer: Correlation with NADPH Oxidases, Angiogenesis, and Apoptosis International Journal of Molecular Sciences 18 2214 10.3390/ijms18102214 29065504 PMC5666894

[b30-turkjbiol-47-5-325] Morgan MJ Liu ZG 2011 Crosstalk of reactive oxygen species and NF-kappaB signaling Cell Research 21 103 115 10.1038/cr.2010.178 21187859 PMC3193400

[b31-turkjbiol-47-5-325] Nelson WG De Marzo AM Isaacs WB 2003 Prostate cancer The New England Journal of Medicine 349 366 381 10.1056/NEJMra021562 12878745

[b32-turkjbiol-47-5-325] Ouyang X DeWeese TL Nelson WG Abate-Shen C 2005 Loss-of-function of Nkx3.1 promotes increased oxidative damage in prostate carcinogenesis Cancer Research 65 6773 6779 10.1158/0008-5472.CAN-05-1948 16061659

[b33-turkjbiol-47-5-325] Rao A Moka N Hamstra DA Ryan CJ 2022 Co-Inhibition of Androgen Receptor and PARP as a Novel Treatment Paradigm in Prostate Cancer-Where Are We Now? Cancers 14 801 10.3390/cancers14030801 35159068 PMC8834038

[b34-turkjbiol-47-5-325] Reddig A Lorenz S Hiemann R Guttek K Hartig R 2015 Assessment of modulated cytostatic drug resistance by automated gammaH2AX analysis Cytometry Part A : The Journal of The International Society for Analytical Cytology 87 724 732 10.1002/cyto.a.22667 25845327

[b35-turkjbiol-47-5-325] Reddig A Voss L Guttek K Roggenbuck D Feist E 2021 Impact of Different JAK Inhibitors and Methotrexate on Lymphocyte Proliferation and DNA Damage Journal of Clinical Medicine 10 1431 10.3390/jcm10071431 33916057 PMC8036268

[b36-turkjbiol-47-5-325] Reuter S Gupta SC Chaturvedi MM Aggarwal BB 2010 Oxidative stress, inflammation, and cancer: how are they linked? Free Radical Biology Medicine 49 1603 1616 10.1016/j.freeradbiomed.2010.09.006 20840865 PMC2990475

[b37-turkjbiol-47-5-325] Rogakou EP Pilch DR Orr AH Ivanova VS Bonner WM 1998 DNA double-stranded breaks induce histone H2AX phosphorylation on serine 139 The Journal of Biological Chemistry 273 5858 5868 10.1074/jbc.273.10.5858 9488723

[b38-turkjbiol-47-5-325] Saito Y Nishio K Ogawa Y Kimata J Kinumi T 2006 Turning point in apoptosis/necrosis induced by hydrogen peroxide Free Radical Research 40 619 630 10.1080/10715760600632552 16753840

[b39-turkjbiol-47-5-325] Takeda M Shirato I Kobayashi M Endou H 1999 Hydrogen peroxide induces necrosis, apoptosis, oncosis and apoptotic oncosis of mouse terminal proximal straight tubule cells Nephron 81 234 238 10.1159/000045282 9933761

[b40-turkjbiol-47-5-325] Tam NN Gao Y Leung YK Ho SM 2003 Androgenic regulation of oxidative stress in the rat prostate: involvement of NAD(P)H oxidases and antioxidant defense machinery during prostatic involution and regrowth The American Journal of Pathology 163 2513 2522 10.1016/S0002-9440(10)63606-1 14633623 PMC1892368

[b41-turkjbiol-47-5-325] Teramoto S Tomita T Matsui H Ohga E Matsuse T 1999 Hydrogen peroxide-induced apoptosis and necrosis in human lung fibroblasts: protective roles of glutathione Japanese Journal of Pharmacology 79 33 40 10.1254/jjp.79.33 10082315

[b42-turkjbiol-47-5-325] Unlu S Kim JW 2022 Emerging Role of PARP Inhibitors in Metastatic Prostate Cancer Current Oncology Reports 24 1619 1631 10.1007/s11912-022-01305-0 35931885

[b43-turkjbiol-47-5-325] Ushio-Fukai M 2009 Compartmentalization of redox signaling through NADPH oxidase-derived ROS Antioxidants Redox Signaling 11 1289 1299 10.1089/ars.2008.2333 18999986 PMC2842113

[b44-turkjbiol-47-5-325] Wang C Sun H Zou R Zhou T Wang S 2015 MDC1 functionally identified as an androgen receptor co-activator participates in suppression of prostate cancer Nucleic Acids Research 43 4893 4908 10.1093/nar/gkv394 25934801 PMC4446443

[b45-turkjbiol-47-5-325] Yuan H Zhang W Du YZ Hu FQ 2010 Ternary nanoparticles of anionic lipid nanoparticles/protamine/DNA for gene delivery International Journal of Pharmaceutics 392 224 231 10.1016/j.ijpharm.2010.03.025 20230883

[b46-turkjbiol-47-5-325] Zhang W van Gent DC Incrocci L van Weerden WM Nonnekens J 2020 Role of the DNA damage response in prostate cancer formation, progression and treatment Prostate Cancer and Prostatic Diseases 23 24 37 10.1038/s41391-019-0153-2 31197228 PMC8076026

